# Epidemiology of urinary tract infections in the Middle East and North Africa, 1990–2021

**DOI:** 10.1186/s41182-025-00692-x

**Published:** 2025-02-05

**Authors:** Fatemeh Amiri, Saeid Safiri, Reza Aletaha, Mark J. M. Sullman, Kamaleddin Hassanzadeh, Ali-Asghar Kolahi, Shahnam Arshi

**Affiliations:** 1 Social Determinants of Health Research Center, Department of Community Medicine, Faculty of Medicine, Tabriz University of Medical Sciences, Tabriz, Iran; 2 Clinical Research Development Unit of Tabriz Valiasr Hospital, Tabriz University of Medical Sciences, Tabriz, Iran; 3 Neurosciences Research Center, Aging Research Institute, Tabriz University of Medical Sciences, Tabriz, Iran; 4 Department of Life and Health Sciences, University of Nicosia, Nicosia, Cyprus; 5 Department of Social Sciences, University of Nicosia, Nicosia, Cyprus; 6 Department of Urology, Faculty of Medicine, Tabriz University of Medical Sciences, Tabriz, Iran; 7 Social Determinants of Health Research Center, Shahid Beheshti University of Medical Sciences, Tehran, Iran

**Keywords:** Urinary tract infections, Incidence, Mortality, Global, Epidemiology

## Abstract

**Objective:**

This research reports the incidence, mortality, and disability-adjusted life years (DALYs) associated with urinary tract infections (UTIs) in the Middle East and North Africa (MENA) region, stratified by sex, age, and Socio-demographic Index (SDI) from 1990 to 2021.

**Methods:**

Data were sourced from the Global Burden of Disease 2021 study, encompassing all 21 countries in the region. Metrics such as absolute counts, age-standardised rates, and percentage changes from 1990 to 2021 are presented with 95% uncertainty intervals (UIs).

**Results:**

In 2019, the MENA region recorded an incidence rate of 4,033.4 per 100,000 (95% UIs: 3,553.7–4,548.7) and 7,687 deaths (95% UIs: 6,663–8,969). The DALY rate was 41.3 per 100,000 (95% UIs: 36.1–47.2), which was highest among older adults, reaching a peak in the 80–89 age range, and increasing with age, particularly from the 50 + age groups. A non-linear relationship was identified between the burden of UTIs and the SDI, with higher-than-expected rates in lower SDI countries such as Syria and Lebanon.

**Conclusion:**

Despite a substantial increase in the total number of UTI cases and DALYs in the region from 1990 to 2021, the age-standardised rates remained stable. The larger burden in lower SDI countries highlights the urgent need for targeted public health interventions. Improving healthcare access and antibiotic stewardship is crucial to mitigating the growing burden of UTIs, particularly among older populations in the region.

**Supplementary Information:**

The online version contains supplementary material available at 10.1186/s41182-025-00692-x.

## Introduction

Urinary tract infections (UTIs) are the most common bacterial infections, affecting any part of the urinary system, including the kidneys, bladder, ureters, and urethra [1]. Annually, approximately 150 million UTI cases occur globally, resulting in direct healthcare costs exceeding $6 billion [2]. In the United States, UTIs account for seven million medical visits each year, with two million related to cystitis and three million patients receiving antibiotic treatment. The economic burden of UTIs in the U.S. is estimated at $3.5 billion, while in Europe, this figure is estimated at €1.5 billion [3, 4]. Globally, UTI cases rose from 252.2 million in 1990 to over 404.6 million in 2019, with the global age-standardised incidence rate rising from 4715.0 per 100,000 people in 1990 to 5229.3 per 100,000 in 2019 [5]. Disability-adjusted life years (DALYs) due to UTIs also surged from 3.1 million in 1990 to nearly 5.2 million by 2019 [5], driven largely by population growth, particularly in low- and middle-income countries [6].

UTIs are more prevalent among women than men due to factors such as the shorter distance between the anus and urethra in women, a shorter female urethra, a more humid environment around the urethra, and the antimicrobial properties of prostate secretions in men [7]. Approximately half of all women have experienced at least one UTI during their life, and despite antibiotic treatment, 20–30% of women develop recurrent UTIs within 3–4 months of an initial infection [3]. Risk factors for UTIs include prior infections, sexual activity, use of condoms, diaphragms, or spermicides, vaginal infections, physical trauma or manipulation, diabetes, obesity, genetic predisposition, and anatomical abnormalities [8].

UTIs cause substantial morbidity, leading to activity limitations, work absences, and time spent in bed, thereby creating a significant healthcare and economic burden, as well as negatively impacting health-related quality of life [9, 10]. Recurrent UTIs are associated with considerable psychological distress, including symptoms of depression and anxiety [11]. To reduce the incidence and burden of UTIs, particularly among women, coordinated efforts from researchers and advocates are necessary to fully quantify the disease burden. This will enable decision-makers to allocate resources more effectively and support evidence-based policy development.

GBD 2021, led by the Institute for Health Metrics and Evaluation, provides comprehensive data on diseases, injuries, and risk factors across 204 countries from 1990 to 2021. However, recent data on UTI prevalence in the Middle East and North Africa (MENA) region are lacking. This research aims to address this gap by presenting updated data on the incidence, deaths, and disability-adjusted life years (DALYs) related to UTIs in all MENA nations from 1990 to 2021, analysed by age, gender, cause, and Socio-demographic Index (SDI).

## Methods

### Overview

The GBD 2021 project evaluated the impact of 371 diseases and injuries across 21 regions, seven super-regions, and 204 nations from 1990 to 2021. Methodological details and advancements since GBD 2019 were discussed in another source [12]. Both fatal and non-fatal estimates are available at: https://vizhub.healthdata.org/gbd-compare/ and https://vizhub.healthdata.org/gbd-results/ [12].

### Case definition and data inputs

A urinary tract infection (UTI) includes conditions such as pyelonephritis, cystitis, urethritis, and other non-specific bacterial infections affecting the urinary system, while excluding asymptomatic bacteriuria. The diagnosis of UTI is confirmed using administrative data and the International Classification of Diseases, 10th Revision (ICD-10) codes. The specific ICD-10 codes used were N10, N10.0, N10.9, N11, N11.0, N11.1, N11.8, N11.9, N12, N12.0, N12.9, N13.6, N15, N15.1, N15.8, N15.9, N16, N16.0-N16.5, N16.8, N30, N30.0-N30.3, N30.8-N30.9, N34, N34.0-N34.3, and N39.0 [13].

The data for this study were sourced from hospital discharge records and claims data from multiple countries. For the GBD 2021 analysis, additional years of claims data were incorporated, enhancing the dataset. Hospital discharge data were also newly integrated to ensure comprehensive coverage [12]. An updated systematic literature review was not undertaken in GBD 2021. The inputs for non-fatal modelling included cause-specific mortality rate estimates derived from our fatal modelling approach, as well as excess mortality rate estimations generated externally to DisMod-MR [12].

The data utilised for estimating mortality from UTIs included vital registration records and verbal autopsy information from the cause of death database. Outliers were identified through a systematic review of data points across all location-years. Data were excluded if they contradicted well-established age or temporal trends. Additionally, cases where garbage code redistribution and noise reduction, combined with small sample sizes, led to implausible cause fractions, were discarded. The criteria for determining outlier status were applied consistently to both vital registration and verbal autopsy data [14].

### Data processing

For incidence data processing, hospital discharge records provided information on encounters, typically including only the primary diagnosis for the visit. Conversely, claims data linked both inpatient and outpatient encounters for individuals, offering primary and secondary diagnoses across all visits. In GBD 2017, an incident case in claims data was defined as any individual with at least one inpatient encounter associated with a relevant ICD code. Hospital discharge data were limited to encounters where the primary diagnosis was relevant and were adjusted for readmissions. In GBD 2019 and 2021, the methodology was updated to capture cases from both inpatient and outpatient settings. An incident case was identified in claims data if the individual had at least one encounter (either inpatient or outpatient) with a relevant ICD code within a year. Hospital discharge data continued to be processed using the primary diagnosis but were adjusted using correction factors derived from claims data. Specifically, the ratio of inpatient claims with a primary UTI diagnosis to all incident UTI cases in claims data was used for these adjustments. In GBD 2021, the correction factor estimation was refined by applying three frequency-placed knots in the age-spline parameter of the MR-BRT analysis. The other data processing steps remained consistent with GBD 2019. Additionally, for the United States, claims data were adjusted for selection bias related to commercial insurance using MR-BRT. In GBD 2021, age was introduced as a covariate to enhance bias adjustment accuracy. Data points with age-standardised incidence rates exceeding two median absolute deviations from the median rate were classified as outliers and excluded from further analysis. Further methodological details are available in the GBD 2021 capstone publication [12].

In GBD 2017, *excess mortality rate* inputs were calculated by linking prevalence data points with corresponding *cause-specific mortality rate* values for the same sex, age, location, and year (by dividing the *cause-specific mortality rate* by prevalence). For short-duration conditions (remission > 1), prevalence was estimated by running an initial model and using the same *cause-specific mortality rate*-to-prevalence ratio. However, this method yielded *excess mortality rate* patterns that were inconsistent with the expected trend of decreasing *excess mortality* in areas with better healthcare access. These unexpected patterns suggested possible discrepancies between *cause-specific mortality rate* estimates and prevalence or incidence measures. To address this, GBD 2019 employed a modelling approach using MR-BRT, which incorporated age, sex, and the Healthcare Access and Quality Index, assigning a negative coefficient to the Healthcare Access and Quality Index. In GBD 2021, the same MR-BRT approach was used to predict *excess mortality rates* across different locations, years, sexes, and for ages 0, 10, 20, and up to 100. These predictions were then used as inputs for the non-fatal modelling [12].

As in previous iterations, the DisMod-MR 2.1 model was utilised to generate estimates by location, sex, age, and year. The model inputs for UTIs included incidence, *cause-specific mortality rate*, and *excess mortality rate*, processed as previously outlined. A prior value was applied to ensure that all cases were resolved within one week, and an upper limit of 0.002 was set for the *excess mortality rate* in individuals aged 0 to 15. Additional information is available in the GBD capstone paper [12].

The estimation approach for UTIs closely mirrors the methods employed in GBD 2019. A standard Causes of Death Ensemble Model (CODEm), incorporating location-level covariates, was utilised to assess deaths attributable to UTIs. Separate models were developed for male and female mortality, with age restrictions for death estimations set from 0 days to 95 years and older. IHME combined separate global models and data-rich models to obtain unadjusted results, which were then adjusted using CoDCorrect and compared to the reference life table to determine the final Years of Life Lost (YLLs) due to UTIs, as outlined in the GBD 2021 appendix section on the Causes of Death database. Additional details are available in the GBD capstone paper [14].

### Severity split and disability weight

The GBD disability weight survey assessments are grounded in lay descriptions of sequelae that highlight key functional impacts and symptoms. UTIs are categorised into mild and moderate severity levels. Mild severity has a disability weight of 0.006 (95% CI: 0.002, 0.012), typically causing low-grade fever and slight discomfort, but no difficulties with everyday activities. In contrast, moderate severity is assigned a disability weight of 0.051 (95% CI: 0.032, 0.074), and is characterised by systemic symptoms such as weakness, aches, and fever, resulting in some challenges with everyday activities [12].

DALY is a widely used metric for assessing the impact of a disorder or disease. It is calculated by summing the years of life lost due to premature mortality (YLLs) and the years lived with disability (YLDs). Additionally, YLDs are determined by multiplying the prevalence within each severity category by the corresponding severity-specific disability weights. To account for uncertainty from multiple sources—including input data, estimates of residual non-sampling errors, and corrections for measurement errors—1,000 samples were drawn during each computation phase. The uncertainty intervals (UIs) were generated using the 2.5th and 97.5th percentiles of the numerically ordered samples.

The association between the SDI and the burden of UTIs, as measured by DALYs, was analysed using smoothing splines. SDI scores range from 0 to 1, with 0 representing the least developed countries and 1 indicating the most developed. These scores are calculated based on the total fertility rate for individuals under 25, the average years of schooling for individuals over 15, and the smoothed gross domestic product per capita over the past decade. R software (version 3.5.2) was employed to plot the incidence rates, age-standardised point prevalence, and YLD rates.

## Results

### The MENA region

In 2021, the estimated incidence of UTIs in the North Africa and Middle East (MENA) region was 25,815,054 cases (95% UI: 22,626,814 to 29,210,790), corresponding to an age-standardised rate of 4,033.4 per 100,000 (95% UI: 3,553.7 to 4,548.7). Between 1990 and 2021, there were no significant changes in the ASRs (Table 1 and S1). In terms of mortality, in 2021, there were 7,687 deaths (95% UI: 6,663 to 8,969) linked to these conditions, yielding an age-standardised rate of 2.3 per 100,000 population (95% UI: 1.9 to 2.7). No significant changes in this rate were noted (Table 1 and S2). In 2021, the MENA region lost 179,393 (95% UI: 155,583 to 203,058) DALYs due to UTIs, resulting in an age-standardised rate of 41.3 per 100,000 population (95% UI: 36.1 to 47.2). There were no significant changes in the age-standardised DALY rate between 1990 and 2021 (Table 1 and S3).

**Table 1:** Incidence, deaths and DALYs due to urinary tract infections in 2021, and the percentage change in the age-standardised rates during the period 1990–2021 (generated from data available from http://ghdx.healthdata.org/gbd-results-tool)

	Incidence (95% UI)			Deaths (95% UI)			DALY (95% UI)		
	Counts (2021)	ASRs (2021)	Pcs in ASRs 1990–2021	Counts (2021)	ASRs (2021)	Pcs in ASRs 1990–2021	Counts (2021)	ASRs (2021)	Pcs in ASRs 1990–2021
North Africa and Middle East	25,815,054 (22,626,814, 29,210,790)	4033.4 (3553.7, 4548.7)	2.1 (− 0.7, 5.3)	7687 (6663, 8969)	2.3 (1.9, 2.7)	2.2 (− 33, 30.5)	179,393 (155,583, 203,058)	41.3 (36.1, 47.2)	− 9.7 (− 35.2, 9.8)
Afghanistan	1,044,474 (892,656, 1,225,341)	3562.6 (3047.7, 4142.8)	− 1.8 (− 12.5, 9.7)	317 (211, 488)	3.6 (2.4, 6.3)	6.2 (− 21.9, 50)	12,015 (7916, 16,561)	79.5 (52.8, 126.4)	− 0.3 (− 26.1, 39.8)
Algeria	1,834,264 (1,585,426, 2,084,776)	4094 (3561.3, 4670.7)	3.1 (− 5.4, 11.9)	627 (499, 784)	2.8 (2.2, 3.5)	5.4 (− 40.3, 69.4)	13,512 (10,706, 16,703)	44.3 (35.8, 54.2)	− 3.1 (− 37.7, 44.8)
Bahrain	64,239 (54,739, 74,071)	3935.9 (3403.4, 4492.7)	− 1.5 (− 10.5, 7.9)	6 (4, 10)	1.7 (1.1, 3.1)	22.4 (− 34.7, 177.4)	154 (111, 224)	25 (16.8, 43.3)	9.5 (− 33.2, 132.2)
Egypt	4,163,108 (3,568,905, 4,845,140)	3912.8 (3384.3, 4517)	2 (− 6.7, 12.5)	292 (191, 379)	0.8 (0.6, 1)	155.5 (7.6, 326.3)	9262 (6690, 11,573)	15.5 (11.2, 19)	100.3 (4.5, 195.4)
Iran	3,723,534 (3,272,014, 4,176,545)	4166.7 (3690.9, 4678.3)	3.1 (0.5, 5.9)	1082 (790, 1786)	1.7 (1.2, 2.8)	− 23 (− 58.3, 3.1)	23,541 (18,722, 33,637)	32.1 (25.2, 47.8)	− 37.1 (− 60.5, − 12.3)
Iraq	1,665,929 (1,432,332, 1,934,288)	3946.8 (3438.4, 4525)	2 (− 6.3, 11.4)	116 (81, 148)	0.7 (0.5, 0.9)	5.4 (− 46.6, 56.3)	3646 (2837, 4558)	14.4 (10.9, 18)	− 11.7 (− 47.5, 20.9)
Jordan	617,656 (493,749, 804,240)	4818.6 (3904.2, 6242.3)	20.9 (3.6, 55.9)	57 (44, 71)	1.2 (0.9, 1.5)	4.2 (− 35.3, 62)	1621 (1281, 2003)	22.1 (17.5, 27.1)	− 6.2 (− 35.9, 35.7)
Kuwait	248,205 (216,491, 286,465)	4799.8 (4255.2, 5463)	9 (0.6, 18.4)	56 (45, 67)	2.6 (2.1, 3.2)	1258.3 (1020.3, 1492.8)	1224 (1020, 1445)	44.5 (36.3, 52.8)	602.1 (447.5, 787.2)
Lebanon	249,748 (218,857, 282,896)	4375.5 (3846.9, 4914.4)	1.7 (− 7.1, 11.5)	349 (273, 455)	5.1 (4, 6.6)	− 12.6 (− 32.1, 13.8)	5118 (4176, 6454)	79.7 (65.5, 99)	− 25.7 (− 42.1, − 6.3)
Libya	308,040 (268,292, 356,114)	4182.8 (3670.5, 4806.5)	5.1 (− 4.5, 16.4)	110 (83, 146)	2.6 (2, 3.4)	46.8 (− 32.1, 163.9)	2717 (1994, 3570)	52.5 (39.5, 68.3)	46 (− 28.7, 143.8)
Morocco	1,525,370 (1,322,416, 1,754,358)	4001.7 (3477.8, 4581.7)	2.9 (− 5.6, 13.1)	785 (571, 1156)	2.9 (2.1, 4.3)	41 (− 18.1, 127.9)	16,911 (12,275, 23,045)	53.5 (39.2, 75.3)	23.2 (− 15.9, 80)
Oman	187,445 (161,239, 216,049)	3784.3 (3304, 4358.9)	5.1 (− 4.6, 16.1)	53 (41, 67)	4.1 (2.9, 5.3)	25.6 (− 33.3, 97.4)	1474 (1130, 1879)	73.1 (56.6, 91.2)	6.5 (− 30.5, 61.5)
Palestine	231,856 (197,407, 269,029)	4457.2 (3883.8, 5129)	1.7 (− 6.8, 11.2)	28 (17, 37)	1.8 (1.1, 2.4)	− 2.7 (− 36.2, 43.1)	684 (479, 887)	28.9 (18.6, 37.2)	− 6.9 (− 35.8, 32.1)
Qatar	136,597 (116,318, 159,872)	4276.2 (3744.9, 4878.8)	5.1 (− 3.2, 14.8)	2 (1, 3)	0.5 (0.3, 0.7)	− 60.6 (− 78.1, 22.9)	128 (90, 181)	9.8 (6.8, 13.3)	− 51.7 (− 71.7, 22.8)
Saudi Arabia	1,572,287 (1,333,205, 1,812,376)	3846.1 (3334.4, 4400.6)	4.7 (− 4.6, 14.5)	683 (525, 864)	6.2 (4.9, 7.8)	17.2 (− 37, 73.8)	20,442 (15,491, 26,482)	106.3 (82.4, 132.8)	8.2 (− 36.6, 57.4)
Sudan	1,623,466 (1,387,477, 1,906,718)	3757.6 (3242.9, 4334.5)	3.5 (− 6.2, 15.2)	324 (225, 486)	2 (1.4, 3.1)	11.6 (− 29.4, 78.3)	10,568 (7374, 14,556)	41.2 (29.3, 59.4)	− 1.2 (− 30.1, 49.7)
Syrian Arab Republic	641,657 (566,780, 758,870)	4514.8 (3990.6, 5274.7)	8.8 (− 1.4, 19.8)	609 (441, 861)	6.8 (4.7, 9.8)	− 20.6 (− 61.6, 30.7)	13,901 (10,367, 18,989)	122.2 (92.1, 168.8)	− 29.3 (− 58.8, 13.3)
Tunisia	537,889 (474,578, 610,431)	4442.1 (3897, 5053.6)	3.4 (− 5.8, 14.2)	231 (166, 333)	2.1 (1.5, 3.1)	20.5 (− 13.7, 72.7)	4373 (3265, 5881)	36.9 (27.6, 49.8)	4.7 (− 25.2, 49.6)
Turkey	3,820,999 (3,318,370, 4,377,880)	4431.2 (3839.3, 5077.5)	4.9 (− 3.2, 14.6)	1722 (1348, 2103)	2.2 (1.7, 2.7)	− 3.1 (− 43.3, 41.6)	30,238 (24,512, 36,545)	36.1 (29.4, 43.4)	− 19.9 (− 47.4, 7.8)
United Arab Emirates	360,524 (301,843, 430,969)	3692.7 (3257, 4307.6)	3.6 (− 4.6, 13)	36 (27, 46)	3 (2.1, 3.9)	− 3.1 (− 49.4, 50.8)	1253 (1009, 1554)	51.1 (38.7, 64.6)	− 11.5 (− 44.5, 30.4)
Yemen	1,233,690 (1,060,553, 1,455,233)	3748.4 (3237.7, 4345.7)	3.4 (− 7.3, 15)	195 (124, 320)	1.8 (1.2, 3.1)	16.4 (− 23.3, 75)	6443 (4194, 9693)	36.1 (24, 57.1)	3.4 (− 28, 46.8)

### Country level

**Fig. 1:**
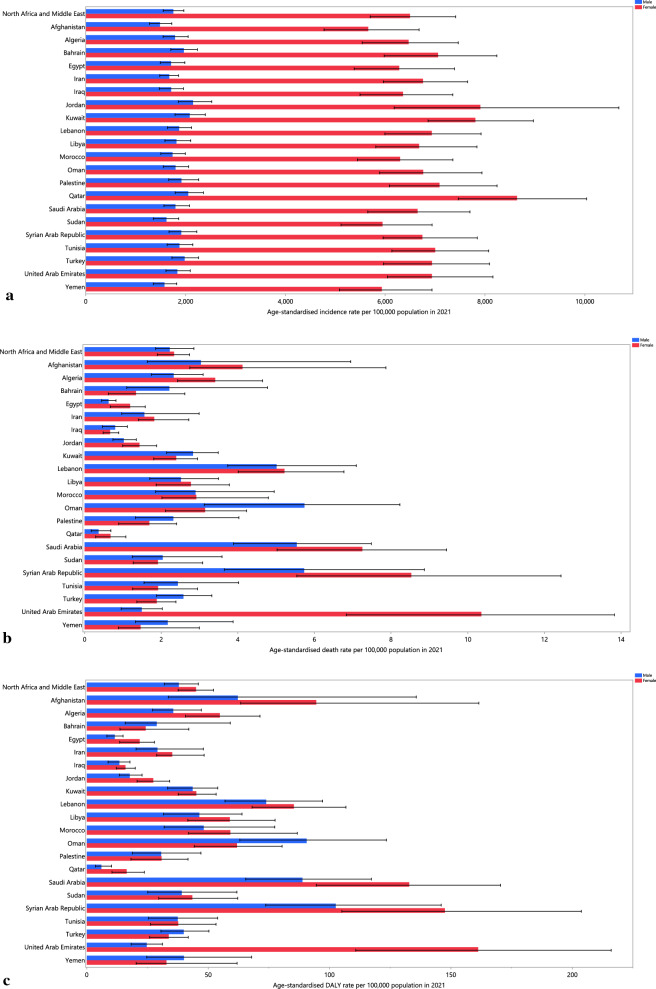
Age-standardised incidence (**a**), deaths (**b**), and DALYs (**c**) for urinary tract infections (per 100,000) in the MENA region in 2021, by sex and country (generated from data available from http://ghdx.healthdata.org/gbd-results-tool)

In 2021, the national age-standardised incidence rates of UTIs in the MENA region varied between 3,562.6 and 4,818.6 cases per 100,000. The largest incidence rates were recorded in Jordan [4,818.6 (95% UI: 3,904.2 to 6,242.3)], Kuwait [4,799.8 (95% UI: 4,255.2 to 5,463.0)], and Turkey [4,431.2 (95% UI: 3,839.3 to 5,077.5)], while the lowest were seen in Afghanistan [3,562.6 (95% UI: 3,047.7 to 4,142.8)], Yemen [3,748.4 (95% UI: 3,237.7 to 4,345.7)], and Sudan [3,757.6 (95% UI: 3,242.9 to 4,334.5)] (Fig. 1A and Table S1).

In 2021, the age-standardised death rates for UTIs in the region ranged from 0.5 to 6.8 per 100,000. The largest rates were recorded in Saudi Arabia [6.2 (95% UI: 4.9 to 7.8)], Lebanon [5.1 (95% UI: 4.0 to 6.6)] and Syria [6.8 (95% UI: 4.7 to 9.8)], while the smallest were found in Qatar [0.5 (95% UI: 0.3 to 0.7)], Kuwait [2.6 (95% UI: 2.1 to 3.2)], and Palestine [1.8 (95% UI: 1.1 to 2.4)] (Fig. 1B and Table S2).

In 2021, the age-standardised DALY rates for UTIs in the region ranged from 9.8 to 122.2 per 100,000. The largest rates were found in Saudi Arabia [106.3 (95% UI: 82.4 to 132.8)], followed by Syria [122.2 (95% UI: 92.1 to 168.8)] and Lebanon [79.7 (95% UI: 65.5 to 99.0)], while the lowest were reported in Qatar [9.8 (95% UI: 6.8 to 13.3)], Kuwait [44.5 (95% UI: 36.3 to 52.8)], and Palestine [28.9 (95% UI: 18.6 to 37.2)] (Fig. 1C and Table S3).

The incidence of UTIs in the region saw a substantial increase between 1990 and 2021. The total number of cases rose from 13.1 million in 1990 to 25.8 million in 2021, although the age-standardised rates exhibited no significant changes. However, trends in age-standardised rates varied across countries. Jordan recorded the highest percentage increase in age-standardised rates, at 20.9% (95% UI: 3.6 to 55.9%), followed by Kuwait at 9.0% (95% UI: 0.6 to 18.4%), while Iran experienced a modest rise of 3.1% (95% UI: 0.5 to 5.9%). The remaining countries had no significant changes in age-standardised rates. Overall, despite the rise in incident cases, the MENA region maintained relatively stable age-standardised rates, with most countries not experiencing significant changes throughout the measurement period (Table S1 and Figure S1).

The total number of deaths attributed to UTIs in MENA rose from 2,969 in 1990 to 7,687 in 2021. While overall changes in age-standardised rates were not significant during this period, Kuwait experienced the most substantial increase at 1,258.3% (95% UI: 1,020.3 to 1,492.8%), followed by Egypt at 155.5% (95% UI: 7.6 to 326.3%). No significant changes were observed in the age-standardised rates of other countries. In summary, although the region experienced a rise in the number of deaths, the age-standardised rates remained relatively stable between 1990 and 2021 (Table S2 and Figure S2).

The total number of DALYs rose from 98,757 in 1990 to 179,393 in 2021, while ASRs showed no significant changes. However, there were notable variations in the percentage changes in ASRs across countries. Kuwait experienced the largest increase, at 602.1% (95% UI: 447.5 to 787.2%), followed by Egypt at 100.3% (95% UI: 4.5 to 195.4%). Conversely, Iran saw the greatest reduction in ASRs, at 37.1% (95% UI: -60.5 to -12.3%), followed by Lebanon at 25.7% (95% UI: -42.1 to -6.3%). The remaining countries did not change significantly over this period. As a whole, the region experienced a rise in the number of DALYs due to UTIs, although the ASRs remained stable in many countries (Table S3 and Figure S3).

### Sex and age patterns

**Fig. 2:**
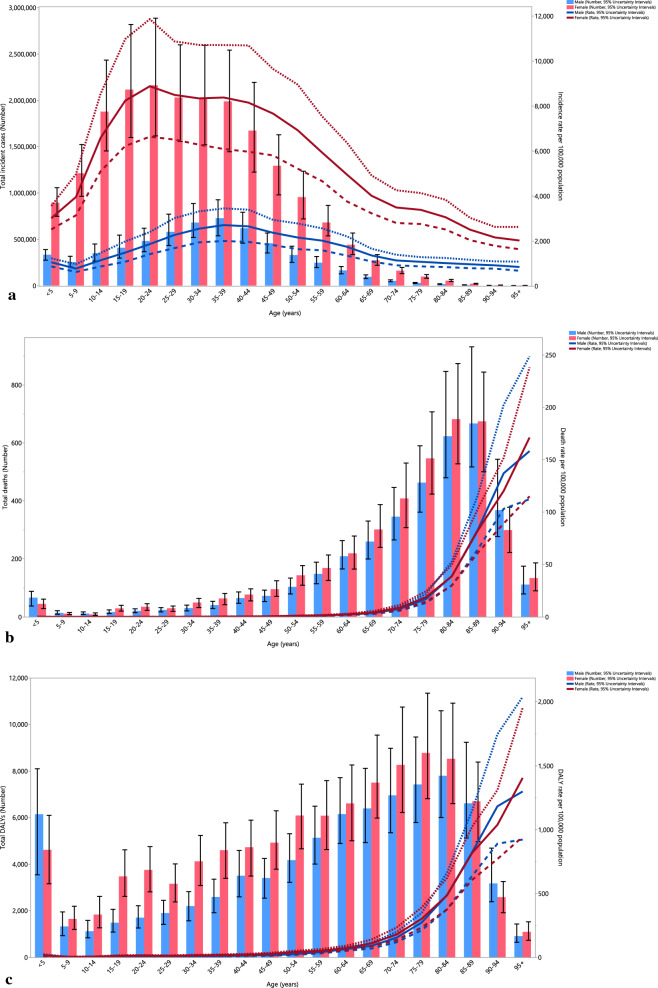
Number of incident cases and incidence rate (**a**), the number of deaths and death rate (**b**), and the total number of DALYs and DALY rate (**c**), for urinary tract infections (per 100,000) in the MENA region, by age and sex in 2021; dotted and dashed lines indicate 95% upper and lower uncertainty intervals, respectively (generated from data available from http://ghdx.healthdata.org/gbd-results-tool)

In 2021, the total number of new UTI cases and incidence rates rose with age, peaking in the 20–24 age range for females and the 35–39 age range for males. Subsequently, the incidence rate gradually declined with increasing age for males, whereas females experienced a sharp drop in the 45 + age range (Fig. 2A). For both sexes, total deaths and death rates decreased slightly up to the 5–9 age range, then rose steadily with age, peaking in the 80–89 age group. A marked decline in total deaths was observed in the 90 + age range for both males and females (Fig. 2B). Similarly, DALY numbers and rates across age groups mirrored that of deaths, decreasing until the 5–9 age range, followed by a gradual increase in older ages, and a sharp decrease after age 85 (Fig. 2C).

In 2021, the total number of incident cases and the incidence rate for UTIs rose with age, reaching a peak in the 20–24 age range for females and the 35–39 age range for males. Subsequently, the incidence rate gradually declined with increasing age for males, while females experienced a sharp decline in the 45 + age group (Fig. 2A). Similarly, total deaths and the death rates for both sexes showed a slight decrease up to the 5–9 age range, followed by an increase with age up to the 80–89 age group. A marked decline in total deaths was observed in the 90 + age group for both sexes (Fig. 2B).

### The MENA/global ratio

**Fig. 3:**
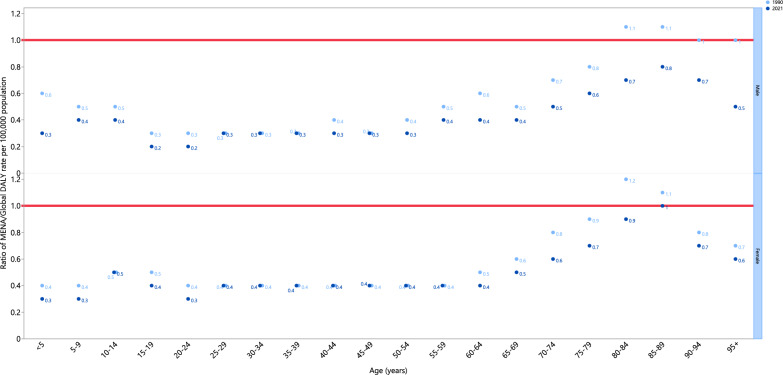
The ratio of the MENA region to the global urinary tract infections DALY rate, according to age group and sex, from 1990 to 2021 (generated from data available from http://ghdx.healthdata.org/gbd-results-tool)

In 2021, the MENA-to-global YLD ratio was consistently equal to or below global rates across all age ranges for both sexes. While both sexes in the 80–89 age group had higher YLD rates than the global average in 1990, this difference had decreased by 2021. For other age groups, the MENA-to-global ratio remained similar between 1990 and 2021 (Fig. 3).

### Association with the SDI

**Fig. 4:**
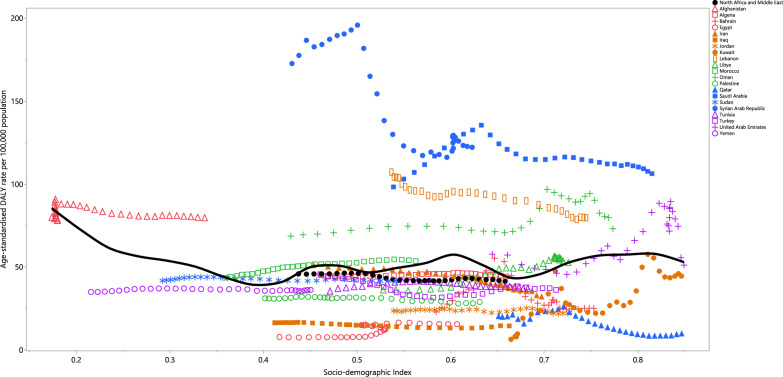
Age-standardised DALY rates of urinary tract infections for the 21 countries and territories in the MENA region in 2021, plotted against the SDI. Expected values based on the SDI and disease rates in all locations are represented by the black line. Each point reflects the observed age-standardised DALY rate for each country (generated from data available from http://ghdx.healthdata.org/gbd-results-tool)

In 2021, the age-standardised DALY rate of UTIs exhibited a non-linear correlation with the Socio-demographic Index (SDI). While the burden decreased up to an SDI of 0.4, it then remained relatively steady. Countries such as Syria, Saudi Arabia, Lebanon, Bahrain, and Oman reported higher-than-expected burdens, while lower-than-expected burdens were observed in Egypt, Qatar, Iraq, Jordan, Palestine, and Kuwait (Fig. 4).

## Discussion

Globally, the burden of UTIs has increased between 1990 and 2021, with the most significant rise found in low-middle SDI countries. Asia had the highest burden globally. The burden is projected to continue its upward trend from 2021 to 2050, with an approximate 40% increase in incidence, prevalence, and DALYs [15]. In line with global data, our study on the MENA region showed an increase in the number of incident cases, deaths and DALYs attributable to UTIs, despite relatively stable age-standardised rates. As the MENA region’s dynamic changes and transitions—including shifts in population size, disease patterns, age demographics, and cultural norms—highlight the need for targeted interventions to address the increasing health burden associated with UTIs, further research and public health initiatives are crucial. Our data provide valuable and updated information that could be instrumental in addressing the challenges posed by UTIs.

The diagnosis of UTIs generally relies on clinical laboratory findings. However, several factors can lead to missed diagnoses, including unclear and non-specific symptoms in children and neonates, subtle symptom presentation in older adults, the presence of multiple pathologies with various symptoms in the elderly, and polypharmacy, which might mask UTI symptoms or distract physicians during diagnosis. Additionally, a high rate of contaminated samples and the tendency of patients to self-medicate without seeking professional care can contribute to diagnostic challenges [16–23]. In developing countries, particularly in the MENA region, inadequate monitoring systems, weak healthcare infrastructure, and improper surveillance strategies further complicate the accurate estimation of UTI incidence [24–26]. According to a meta-analysis by Mengistu et al., the overall pooled incidence rate of UTIs was 1.6%, with a decreasing trend over time [27]. Their findings contrast with the GBD results. This difference may stem from the limited scope of Mengistu's study, which included only 38 studies across 26 countries. Additionally, Mengistu's analysis did not incorporate ASR rates, which may have affected the precision in estimating the global burden of the disease. Furthermore, UTI-causing pathogens have developed significant antibiotic resistance, with increasingly diverse and potent resistance types contributing to the growing burden of UTIs [28–30].

Antibiotic resistance presents a critical challenge in the MENA region, with multidrug-resistant pathogens significantly complicating treatment efforts. Among the most concerning resistant strains are extended-spectrum beta-lactamase (ESBL)-producing *Escherichia coli* and *Klebsiella pneumoniae*, which are notably prevalent throughout the region. These strains contribute to increased treatment failure rates, longer hospital stays, and increased mortality. The emergence of carbapenem-resistant Enterobacteriaceae (CRE) and other resistant organisms further complicates treatment, making infections increasingly difficult to manage. Addressing these issues requires robust antimicrobial stewardship programmes, improved laboratory capacities for pathogen identification, and coordinated regional efforts to reduce antibiotic misuse [31–34].

In line with global findings [15] and previous research [5], our study indicates a rise in the total number of incident UTI cases in the region, from 13.1 million in 1990 to 25.8 million in 2021. Although the number of cases has approximately doubled, the ASR of incidence has remained relatively stable, suggesting that the incidence rate per capita has not changed drastically. Several factors may contribute to this trend, including improved diagnosis and reporting mechanisms, population growth, and enhanced healthcare access in some parts of the region.

The observed decline in UTI incidence among women aged 45 and older can be attributed to several factors. Postmenopausal changes, such as alterations in vaginal flora and decreased oestrogen levels, contribute to reduced susceptibility to UTIs. Additionally, lifestyle modifications—including reduced sexual activity and changes in healthcare-seeking behaviour—may also play a role in this decline. However, it is important to note that the risk of severe UTI-related complications continues to be elevated in older women, particularly those with underlying chronic conditions such as diabetes and kidney disease [35–37].

However, it is important to recognise the significant disparities between countries within the MENA region. For example, countries such as Jordan, Kuwait, and Turkey have the largest age-standardised incidence rates, while Afghanistan, Yemen, and Sudan had much lower rates. Differences in healthcare infrastructure, access to clean water and sanitation, and socio-political stability may contribute to variations in the UTI burden. Additionally, diagnostic capabilities and healthcare accessibility might confound the results. Inadequate healthcare infrastructure, ongoing conflict, and limited access to clean water and sanitation can hinder accurate diagnosis and reporting of diseases, including UTIs [38–40]. The lack of access to primary healthcare services, particularly in rural areas, may also result in underreporting of UTI cases in these countries. Furthermore, the prevalence of risk factors such as malnutrition, poverty, and poor living conditions exacerbates the burden of infectious diseases, including UTIs, while increasing the rate of self-medication [41–44]. However, it is important to consider increased exposure to risk factors, such as the higher rates of diabetes in Kuwait [45] and Turkey [46], although this does not necessarily predict outcomes, as will be discussed in the mortality and DALYs sections.

Countries with higher reported incidence rates may benefit from better healthcare and reporting systems, but face increasing pressure to manage the growing number of UTI cases. This underscores the need for targeted interventions addressing specific risk factors and healthcare challenges in the MENA region. Improving access to clean water, functional healthcare systems, and awareness of UTI prevention and treatment is crucial.

A concerning finding of our study is the significant increase in UTI-related deaths in the MENA region, rising from 2,969 in 1990 to 7,687 in 2021, representing a greater than twofold increase. While the age-standardised mortality rate has remained relatively stable overall, alarming trends are observed in some countries. Kuwait, in particular, experienced a dramatic 1,258.3% rise in UTI-related mortality, while Egypt reported a 155.5% increase. Factors contributing to this trend include healthcare access issues, the rise in antibiotic-resistant infections, changing pathogen strains, increased hospitalisation and catheterisation rates, an aging population with increased urosepsis rates, and challenges in managing UTI complications [47–53]. In Kuwait, the significant rise in mortality may also reflect changes in healthcare delivery or reporting practices, as well as an increasing prevalence of chronic conditions such as obesity and diabetes that predispose individuals to severe UTI-related complications [54, 55].

The growing burden of antibiotic resistance in the MENA region is a critical factor in UTI-related mortality. Al-Orphaly et al. have highlighted the increasing prevalence of multidrug-resistant bacterial strains, including those responsible for UTIs, across the region [56]. The emergence of resistant strains, such as extended-spectrum beta-lactamase (ESBL)-producing *Escherichia coli* and *Klebsiella pneumoniae*, has made UTI treatment more challenging, leading to higher rates of treatment failure, prolonged hospital stays, and increased mortality [57]. The lack of effective antimicrobial stewardship programmes and the overuse of antibiotics in some MENA countries further exacerbate the problem of resistance, contributing to the rising mortality rates observed in countries like Kuwait and Egypt [58–61].

In contrast, some countries in the region have managed to maintain stable or declining mortality rates. For example, Iran and Lebanon have experienced significant decreases in UTI-related ASRs for mortality. These declines may be attributed to improvements in healthcare delivery, better management of UTI complications, and more effective public health interventions aimed at reducing the incidence and severity of UTIs [62, 63]. In Iran, the health system underwent a series of changes over several decades. Establishing rural healthcare centres and rural insurance programmes across the country ensures that everyone in the country, regardless of socioeconomic status, geographical location, ethnicity, or sex, has access to affordable healthcare and essential medicine [64–66]. These countries can serve as models for other nations in the region, demonstrating the importance of strong healthcare systems, effective antibiotic stewardship programmes, and public health campaigns aimed at preventing UTIs and managing complications.

Our data also reveal important insights into the age and sex distribution of UTI burden in the MENA region. The findings show that UTI incidence rates peak in females aged 20–24, which is consistent with global patterns where women of reproductive age are at higher risk of developing UTIs [15]. This increased risk in women can be attributed to several factors, including anatomical differences, hormonal changes, and sexual activity, which make them more susceptible to UTIs during the reproductive years [67–69].

The decline in UTI incidence among older women, particularly after menopause, suggests the potential impact of alterations in vaginal flora, decreased oestrogen levels, and changes in the urinary tract. However, it is important to note that older women remain at risk of developing more severe UTI-related complications, especially if they have underlying chronic conditions such as diabetes or kidney disease [70, 71].

In 2021, the incidence of UTIs in males was peaked in the 35–39 age range. In middle-aged men, the risk of developing UTIs increases, primarily due to factors such as urinary retention and infection resulted from benign prostatic hyperplasia (BPH), other urinary tract abnormalities, and urinary stone disease [72, 73]. The decline in deaths and DALYs in the 90 + age group in both sexes may reflect, higher mortality rates in younger elderly individuals, resulting in fewer individuals surviving into the 90 + age group.

Given the sex differences in UTI burden, sex-specific public health interventions could be beneficial. Public health efforts should focus on promoting good hygiene practices, safe sexual behaviour, and early medical consultations to prevent UTI complications in high-risk groups. For older adults, managing risk factors such as diabetes and kidney disease control could help prevent complications like sepsis and septic shock.

The association between UTIs and SDI revealed a non-linear correlation. In lower SDI countries like Syria and Lebanon, the burden was higher than expected. Damaged healthcare systems, particularly in conflict-affected areas, can negatively impact health outcomes for UTIs and other conditions. Higher SDI countries like Kuwait and Qatar reported lower-than-expected burdens, consistent with studies showing that access to clean water, sanitation, and healthcare in high SDI regions protects against UTI-related complications [74]. Additionally, low-resource settings affect health-seeking behaviours for UTI, influencing disease epidemiology and outcomes [75].

Education plays a crucial role in reducing the UTI burden. Women who are informed about hygiene practices, sexual health, and the importance of seeking medical care are more likely to prevent and treat UTIs effectively [63, 76]. Public health campaigns that educate women and communities about UTI prevention can significantly reduce the disease burden in the region.

With the rising burden of UTIs in MENA, improving healthcare access, particularly in poor and rural areas, and strengthening healthcare systems for better UTI management are essential. Governments should also invest in public health campaigns promoting UTI prevention awareness, especially among women and older adults. Educational programmes focusing on hygiene practices, sexual health, and seeking medical care can help reduce UTI incidence and prevent complications. Additionally, policymakers should consider implementing programmes that provide financial support for individuals who cannot afford healthcare services, as economic barriers to healthcare access significantly contribute to the UTI burden in the region.

Antimicrobial stewardship must also be a top priority for MENA countries to address the growing threat of antibiotic resistance. National health policies should include regulations limiting over-the-counter antibiotics sales and promoting responsible antibiotics use by physicians. Furthermore, improving access to clean water and sanitation is essential for preventing UTIs and improving health outcomes, particularly in low-income and conflict-affected areas.

### Strengths and limitations

Our study provides a comprehensive analysis of UTI trends in the MENA region over 31 years, using data from the Global Burden of Disease (GBD) 2021 Study, a robust and widely recognised source of global health data. It highlights critical regional disparities and identifies key risk factors, thereby providing actionable insights for public health interventions. However, like most GBD studies, our study also has several limitations. First, while our study provides a comprehensive overview of UTI trends in the MENA region, it did not include subgroup analyses and sensitivity analyses due to data and methodological constraints. Future research could incorporate these analyses to enhance the understanding of temporal trends and variability across subpopulations. Second, our reliance on GBD data means that the findings depend on the quality and availability of underlying data sources, which can vary significantly across countries in the MENA region. Developing countries often lack adequate infrastructure to accurately capture and report the disease burden. In regions with limited data, we employed advanced methods to compare with similar regions and address gaps, but this remains a notable limitation of our study. Third, there are potential biases in estimating incidence and mortality rates, as weaker health information systems in some countries may lead to underreporting or misclassification of cases.

## Conclusion

The total number of incident cases, deaths, and DALYs due to UTIs in the MENA region has increased significantly between 1990 and 2021, despite relatively stable age-standardised rates. This upward trend is likely influenced by the region’s demographic and epidemiological transitions, rising antibiotic resistance, and increased exposure to risk factors, highlighting the growing public health challenge posed by UTIs. To address this, MENA countries should prioritise enhancing healthcare access, promoting hygiene and preventive measures, addressing antibiotic resistance, and investing in social determinants of health**—**such as clean water, sanitation, and education. These multifaceted efforts could help reduce the burden of UTIs and improve overall health in the region.

## Supplementary Information

Supplementary material 1.

## Data Availability

The data used for these analyses are all publicly available at: https://vizhub.healthdata.org/gbd-results/.
