# Supplementary material for: Epidemiology of urinary tract infections in the Middle East and North Africa, 1990–2021
Source: Trop Med Health. 2025 Feb 5;53:16. doi: 10.1186/s41182-025-00692-x (PMC11796260; doi:10.1186/s41182-025-00692-x)
Supplement: Supplementary file 1 — Supplementary material 1. [file 41182_2025_692_MOESM1_ESM.pdf]

Table S1: Incidence of urinary tract infections and interstitial nephritis in 1990 and 2021, and the percentage change in age-standardised rates (ASRs) per 100,000 in the Middle East North and Africa region (Generated from data available from http://ghdx.healthdata.org/gbd-results-tool)					
	1990		2021		Percentage change in ASRs per 100,000
	No (95% UI)	ASRs per 100,000 (95% UI)	No (95% UI)	ASRs per 100,000 (95% UI)	
**North Africa and Middle East**	**13078912 (11488246 , 14930562)**	**3951.7 (3503.3 , 4421.4)**	**25815054 (22626814 , 29210790)**	**4033.4 (3553.7 , 4548.7)**	**2.1 (-0.7 , 5.3)**
**Afghanistan**	**327254 (278552 , 387745)**	**3627.2 (3119.7 , 4233.1)**	**1044474 (892656 , 1225341)**	**3562.6 (3047.7 , 4142.8)**	**-1.8 (-12.5 , 9.7)**
**Algeria**	**969482 (833938 , 1124297)**	**3970.4 (3447.6 , 4537.8)**	**1834264 (1585426 , 2084776)**	**4094 (3561.3 , 4670.7)**	**3.1 (-5.4 , 11.9)**
**Bahrain**	**21227 (18294 , 24222)**	**3994 (3544.9 , 4519.5)**	**64239 (54739 , 74071)**	**3935.9 (3403.4 , 4492.7)**	**-1.5 (-10.5 , 7.9)**
**Egypt**	**2086443 (1818059 , 2417257)**	**3835.7 (3355.3 , 4405.6)**	**4163108 (3568905 , 4845140)**	**3912.8 (3384.3 , 4517)**	**2 (-6.7 , 12.5)**
**Iran**	**2227842 (1956461 , 2531044)**	**4041.4 (3576.5 , 4534.7)**	**3723534 (3272014 , 4176545)**	**4166.7 (3690.9 , 4678.3)**	**3.1 (0.5 , 5.9)**
**Iraq**	**684953 (593824 , 789159)**	**3869.3 (3369 , 4407.8)**	**1665929 (1432332 , 1934288)**	**3946.8 (3438.4 , 4525)**	**2 (-6.3 , 11.4)**
**Jordan**	**145914 (125926 , 168028)**	**3984.8 (3496 , 4453)**	**617656 (493749 , 804240)**	**4818.6 (3904.2 , 6242.3)**	**20.9 (3.6 , 55.9)**
**Kuwait**	**80615 (68759 , 91755)**	**4405.4 (3852 , 4971.6)**	**248205 (216491 , 286465)**	**4799.8 (4255.2 , 5463)**	**9 (0.6 , 18.4)**
**Lebanon**	**127429 (111440 , 145314)**	**4300.9 (3789.3 , 4882.5)**	**249748 (218857 , 282896)**	**4375.5 (3846.9 , 4914.4)**	**1.7 (-7.1 , 11.5)**
**Libya**	**166444 (143033 , 195182)**	**3980.1 (3502.9 , 4586.9)**	**308040 (268292 , 356114)**	**4182.8 (3670.5 , 4806.5)**	**5.1 (-4.5 , 16.4)**
**Morocco**	**973133 (843766 , 1109601)**	**3889.9 (3390.5 , 4377.7)**	**1525370 (1322416 , 1754358)**	**4001.7 (3477.8 , 4581.7)**	**2.9 (-5.6 , 13.1)**
**Oman**	**71529 (62158 , 82154)**	**3601.4 (3196.2 , 4132.1)**	**187445 (161239 , 216049)**	**3784.3 (3304 , 4358.9)**	**5.1 (-4.6 , 16.1)**
**Palestine**	**84270 (72861 , 96989)**	**4384.3 (3866.3 , 5011.5)**	**231856 (197407 , 269029)**	**4457.2 (3883.8 , 5129)**	**1.7 (-6.8 , 11.2)**
**Qatar**	**19272 (16857 , 22338)**	**4068.3 (3611.3 , 4721.6)**	**136597 (116318 , 159872)**	**4276.2 (3744.9 , 4878.8)**	**5.1 (-3.2 , 14.8)**
**Saudi Arabia**	**588011 (503199 , 674737)**	**3674.1 (3189.7 , 4144.6)**	**1572287 (1333205 , 1812376)**	**3846.1 (3334.4 , 4400.6)**	**4.7 (-4.6 , 14.5)**
**Sudan**	**688200 (595398 , 801012)**	**3631.9 (3159.5 , 4168.6)**	**1623466 (1387477 , 1906718)**	**3757.6 (3242.9 , 4334.5)**	**3.5 (-6.2 , 15.2)**

**Syrian Arab Republic**	**502302 (439448 , 572239)**	**4151.2 (3670.8 , 4759.5)**	**641657 (566780 , 758870)**	**4514.8 (3990.6 , 5274.7)**	**8.8 (-1.4 , 19.8)**
**Tunisia**	**356360 (308391 , 411710)**	**4294.2 (3760.2 , 4911.6)**	**537889 (474578 , 610431)**	**4442.1 (3897 , 5053.6)**	**3.4 (-5.8 , 14.2)**
**Turkey**	**2435283 (2129918 , 2802579)**	**4223.7 (3719.8 , 4819.1)**	**3820999 (3318370 , 4377880)**	**4431.2 (3839.3 , 5077.5)**	**4.9 (-3.2 , 14.6)**
**United Arab Emirates**	**70430 (60350 , 80034)**	**3565.8 (3114.9 , 4009.1)**	**360524 (301843 , 430969)**	**3692.7 (3257 , 4307.6)**	**3.6 (-4.6 , 13)**
**Yemen**	**445365 (374525 , 522352)**	**3626.7 (3090 , 4214.1)**	**1233690 (1060553 , 1455233)**	**3748.4 (3237.7 , 4345.7)**	**3.4 (-7.3 , 15)**

Table S2: Deaths from urinary tract infections in 1990 and 2021, and the percentage change in age-standardised rates (ASRs) per 100,000 in the Middle East and North Africa region (Generated from data available from http://ghdx.healthdata.org/gbd-results-tool)					
	1990		2021		Percentage change in ASRs per 100,000
	No (95% UI)	ASRs per 100,000 (95% UI)	No (95% UI)	ASRs per 100,000 (95% UI)	
North Africa and Middle East	2969 (2402 , 4484)	2.2 (1.7 , 3.7)	7687 (6663 , 8969)	2.3 (1.9 , 2.7)	2.2 (-33 , 30.5)
Afghanistan	197 (135 , 360)	3.4 (2.3 , 6.7)	317 (211 , 488)	3.6 (2.4 , 6.3)	6.2 (-21.9 , 50)
Algeria	184 (124 , 320)	2.6 (1.7 , 4.8)	627 (499 , 784)	2.8 (2.2 , 3.5)	5.4 (-40.3 , 69.4)
Bahrain	1 (1 , 2)	1.4 (0.8 , 2.4)	6 (4 , 10)	1.7 (1.1 , 3.1)	22.4 (-34.7 , 177.4)
Egypt	51 (35 , 96)	0.3 (0.2 , 0.6)	292 (191 , 379)	0.8 (0.6 , 1)	155.5 (7.6 , 326.3)
Iran	465 (380 , 655)	2.2 (1.7 , 3.5)	1082 (790 , 1786)	1.7 (1.2 , 2.8)	-23 (-58.3 , 3.1)
Iraq	54 (39 , 88)	0.7 (0.5 , 1.1)	116 (81 , 148)	0.7 (0.5 , 0.9)	5.4 (-46.6 , 56.3)
Jordan	12 (9 , 16)	1.2 (0.9 , 1.8)	57 (44 , 71)	1.2 (0.9 , 1.5)	4.2 (-35.3 , 62)
Kuwait	1 (1 , 1)	0.2 (0.2 , 0.2)	56 (45 , 67)	2.6 (2.1 , 3.2)	1258.3 (1020.3 , 1492.8)
Lebanon	98 (80 , 124)	5.9 (4.6 , 7.7)	349 (273 , 455)	5.1 (4 , 6.6)	-12.6 (-32.1 , 13.8)
Libya	31 (20 , 62)	1.8 (1.1 , 3.7)	110 (83 , 146)	2.6 (2 , 3.4)	46.8 (-32.1 , 163.9)
Morocco	258 (166 , 493)	2.1 (1.2 , 4.4)	785 (571 , 1156)	2.9 (2.1 , 4.3)	41 (-18.1 , 127.9)
Oman	19 (14 , 27)	3.3 (2.3 , 4.7)	53 (41 , 67)	4.1 (2.9 , 5.3)	25.6 (-33.3 , 97.4)
Palestine	12 (8 , 20)	1.9 (1.2 , 3.2)	28 (17 , 37)	1.8 (1.1 , 2.4)	-2.7 (-36.2 , 43.1)
Qatar	1 (0 , 1)	1.2 (0.4 , 2.4)	2 (1 , 3)	0.5 (0.3 , 0.7)	-60.6 (-78.1 , 22.9)
Saudi Arabia	246 (177 , 366)	5.3 (3.8 , 8.4)	683 (525 , 864)	6.2 (4.9 , 7.8)	17.2 (-37 , 73.8)
Sudan	153 (105 , 268)	1.8 (1 , 3.7)	324 (225 , 486)	2 (1.4 , 3.1)	11.6 (-29.4 , 78.3)

**Syrian Arab Republic**	**403 (313 , 584)**	**8.5 (6.4 , 13.1)**	**609 (441 , 861)**	**6.8 (4.7 , 9.8)**	**-20.6 (-61.6 , 30.7)**
**Tunisia**	**65 (48 , 99)**	**1.7 (1.3 , 2.8)**	**231 (166 , 333)**	**2.1 (1.5 , 3.1)**	**20.5 (-13.7 , 72.7)**
**Turkey**	**640 (489 , 986)**	**2.2 (1.7 , 3.6)**	**1722 (1348 , 2103)**	**2.2 (1.7 , 2.7)**	**-3.1 (-43.3 , 41.6)**
**United Arab Emirates**	**9 (6 , 14)**	**3.1 (2.1 , 4.9)**	**36 (27 , 46)**	**3 (2.1 , 3.9)**	**-3.1 (-49.4 , 50.8)**
**Yemen**	**68 (45 , 121)**	**1.5 (0.9 , 3.4)**	**195 (124 , 320)**	**1.8 (1.2 , 3.1)**	**16.4 (-23.3 , 75)**

Table S3: DALYs due to urinary tract infections in 1990 and 2021, and the percentage change in age-standardised rates (ASRs) per 100,000 in the Middle East and North Africa region (Generated from data available from http://ghdx.healthdata.org/gbd-results-tool)					
	1990		2021		Percentage change in ASRs per 100,000
	No (95% UI)	ASRs per 100,000 (95% UI)	No (95% UI)	ASRs per 100,000 (95% UI)	
**North Africa and Middle East**	**98757 (84415 , 126863)**	**45.8 (37.7 , 66.6)**	**179393 (155583 , 203058)**	**41.3 (36.1 , 47.2)**	**-9.7 (-35.2 , 9.8)**
**Afghanistan**	**6321 (4616 , 9652)**	**79.8 (56.5 , 138.2)**	**12015 (7916 , 16561)**	**79.5 (52.8 , 126.4)**	**-0.3 (-26.1 , 39.8)**
**Algeria**	**6163 (4627 , 8811)**	**45.7 (31.8 , 75)**	**13512 (10706 , 16703)**	**44.3 (35.8 , 54.2)**	**-3.1 (-37.7 , 44.8)**
**Bahrain**	**39 (27 , 58)**	**22.9 (14.9 , 37.8)**	**154 (111 , 224)**	**25 (16.8 , 43.3)**	**9.5 (-33.2 , 132.2)**
**Egypt**	**2799 (2047 , 3969)**	**7.7 (5.6 , 12.8)**	**9262 (6690 , 11573)**	**15.5 (11.2 , 19)**	**100.3 (4.5 , 195.4)**
**Iran**	**19916 (15530 , 24438)**	**51 (42.4 , 70)**	**23541 (18722 , 33637)**	**32.1 (25.2 , 47.8)**	**-37.1 (-60.5 , -12.3)**
**Iraq**	**1955 (1487 , 2662)**	**16.3 (12.2 , 23.9)**	**3646 (2837 , 4558)**	**14.4 (10.9 , 18)**	**-11.7 (-47.5 , 20.9)**
**Jordan**	**441 (355 , 555)**	**23.6 (18.2 , 32.6)**	**1621 (1281 , 2003)**	**22.1 (17.5 , 27.1)**	**-6.2 (-35.9 , 35.7)**
**Kuwait**	**77 (57 , 101)**	**6.3 (5.1 , 7.7)**	**1224 (1020 , 1445)**	**44.5 (36.3 , 52.8)**	**602.1 (447.5 , 787.2)**
**Lebanon**	**2256 (1854 , 2724)**	**107.2 (87.5 , 132.5)**	**5118 (4176 , 6454)**	**79.7 (65.5 , 99)**	**-25.7 (-42.1 , -6.3)**
**Libya**	**877 (637 , 1371)**	**35.9 (24.3 , 62.8)**	**2717 (1994 , 3570)**	**52.5 (39.5 , 68.3)**	**46 (-28.7 , 143.8)**
**Morocco**	**7778 (5866 , 11027)**	**43.5 (30.3 , 74.7)**	**16911 (12275 , 23045)**	**53.5 (39.2 , 75.3)**	**23.2 (-15.9 , 80)**
**Oman**	**656 (486 , 858)**	**68.6 (49.1 , 95)**	**1474 (1130 , 1879)**	**73.1 (56.6 , 91.2)**	**6.5 (-30.5 , 61.5)**
**Palestine**	**309 (215 , 430)**	**31 (20.7 , 47.7)**	**684 (479 , 887)**	**28.9 (18.6 , 37.2)**	**-6.9 (-35.8 , 32.1)**
**Qatar**	**26 (14 , 36)**	**20.2 (7.8 , 34.1)**	**128 (90 , 181)**	**9.8 (6.8 , 13.3)**	**-51.7 (-71.7 , 22.8)**
**Saudi Arabia**	**7541 (5595 , 10359)**	**98.2 (71.5 , 144.8)**	**20442 (15491 , 26482)**	**106.3 (82.4 , 132.8)**	**8.2 (-36.6 , 57.4)**
**Sudan**	**6258 (4744 , 8239)**	**41.7 (29.1 , 70.2)**	**10568 (7374 , 14556)**	**41.2 (29.3 , 59.4)**	**-1.2 (-30.1 , 49.7)**

**Syrian Arab Republic**	**12603 (9837 , 16051)**	**172.7 (132.6 , 245.9)**	**13901 (10367 , 18989)**	**122.2 (92.1 , 168.8)**	**-29.3 (-58.8 , 13.3)**
**Tunisia**	**1963 (1571 , 2614)**	**35.3 (27.3 , 50.8)**	**4373 (3265 , 5881)**	**36.9 (27.6 , 49.8)**	**4.7 (-25.2 , 49.6)**
**Turkey**	**17378 (13958 , 23435)**	**45.1 (35.8 , 65.3)**	**30238 (24512 , 36545)**	**36.1 (29.4 , 43.4)**	**-19.9 (-47.4 , 7.8)**
**United Arab Emirates**	**342 (239 , 541)**	**57.7 (40.1 , 85.9)**	**1253 (1009 , 1554)**	**51.1 (38.7 , 64.6)**	**-11.5 (-44.5 , 30.4)**
**Yemen**	**3006 (2216 , 4157)**	**34.9 (23 , 64)**	**6443 (4194 , 9693)**	**36.1 (24 , 57.1)**	**3.4 (-28 , 46.8)**

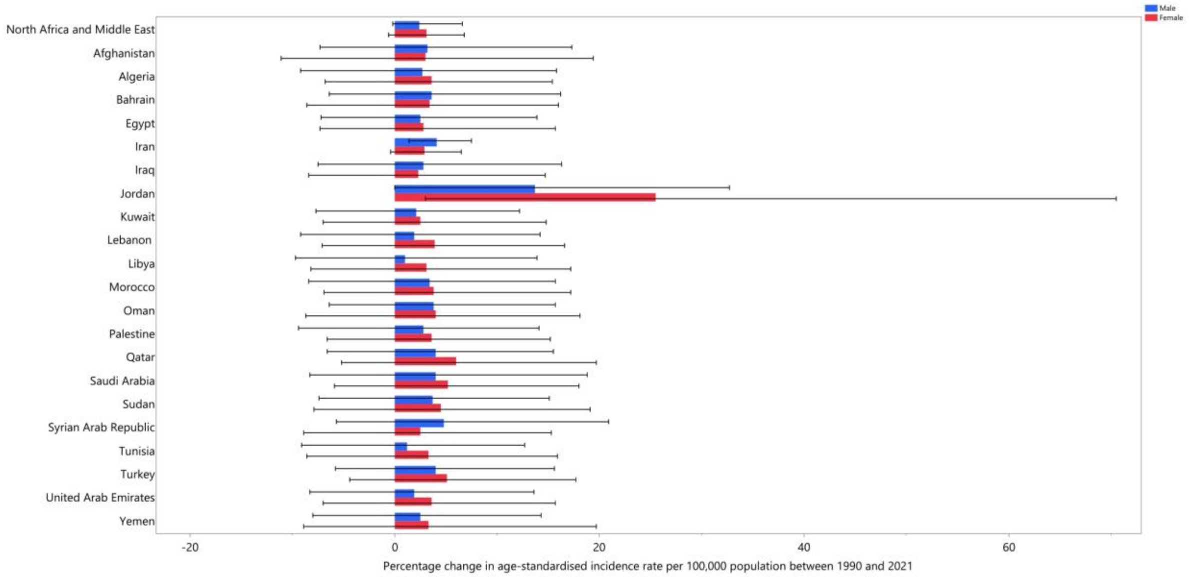

Figure S1: Percentage change in the age-standardised incidence rate of urinary tract infections (per 100,000) in the Middle East and North Africa region from 1990 to 2021, by sex and country.(Data generated from http://ghdx.healthdata.org/gbd-results-tool).

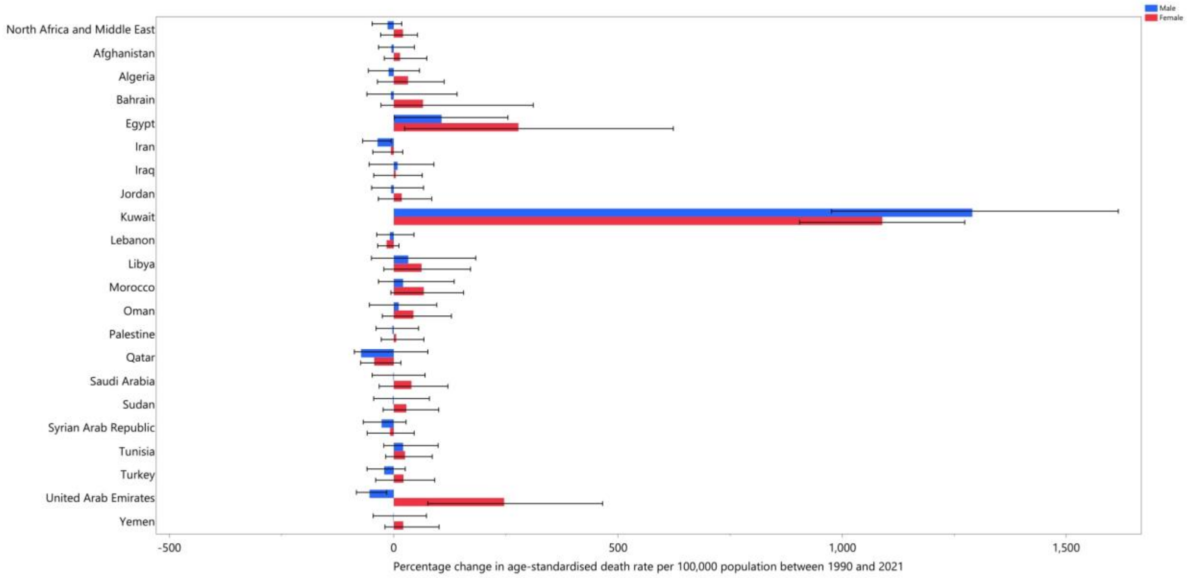

Figure S2: Percentage change in the age-standardised death rate of urinary tract infections (per 100,000) in the Middle East and North Africa region from 1990 to 2021, by sex and country.(Data generated from http://ghdx.healthdata.org/gbd-results-tool).

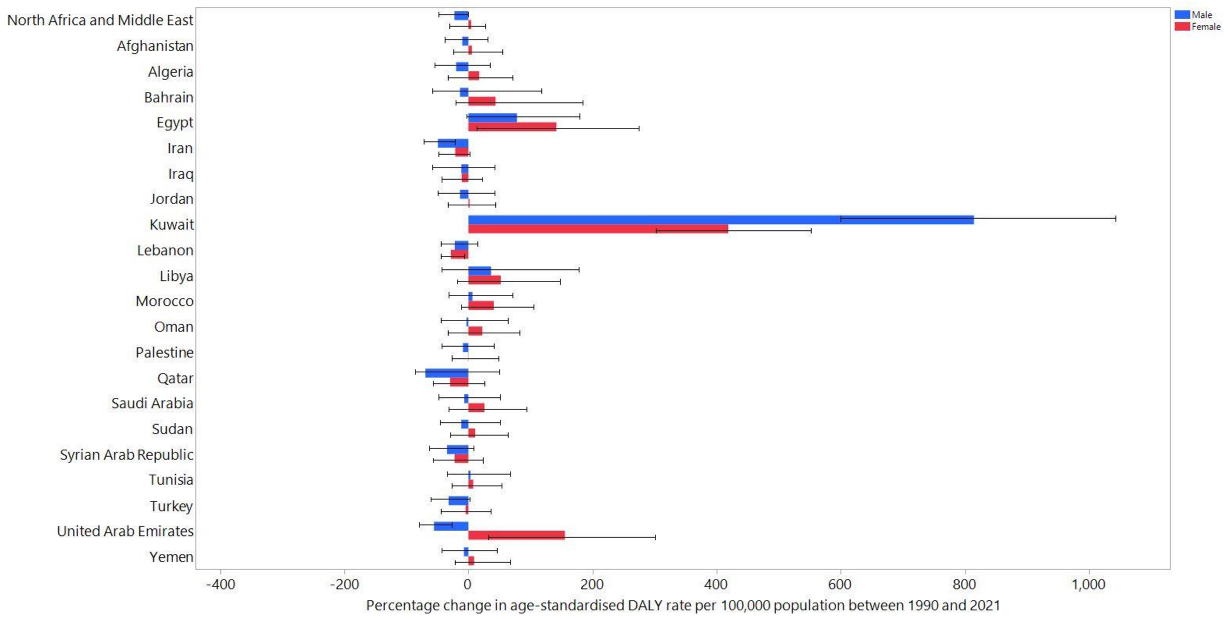

Figure S3: Percentage change in the age-standardised DALY rate of urinary tract infections (per 100,000) in the Middle East and North Africa region from 1990 to 2021, by sex and country. DALY=disability adjusted life years.(Data generated from http://ghdx.healthdata.org/gbd-results-tool).
